# Koninginins N-Q, Polyketides from the Endophytic Fungus *Trichoderma koningiopsis* Harbored in *Panax notoginseng*

**DOI:** 10.1007/s13659-015-0085-z

**Published:** 2016-01-11

**Authors:** Kai Liu, Ya-Bin Yang, Jin-Lian Chen, Cui-Ping Miao, Qiang Wang, Hao Zhou, You-Wei Chen, Yi-Qing Li, Zhong-Tao Ding, Li-Xing Zhao

**Affiliations:** Yunnan Institute of Microbiology, School of Life Science, Yunnan University, Kunming, 650091 People’s Republic of China; Key Laboratory of Medicinal Chemistry for Natural Resource, Ministry of Education, School of Chemical Science and Technology, Yunnan University, Kunming, 650091 People’s Republic of China; School of Energy and Environment Science, Yunnan Normal University, Kunming, 650092 People’s Republic of China

**Keywords:** *Trichoderma koningiopsis*, Polyketide, Antifungal activity, Bioactivity assay, Koninginin

## Abstract

**Abstract:**

Four new fungal polyketides named koninginins N-Q (**1**–**4**), together with four known analogues (**5**–**8**), were isolated from the endophytic fungus *Trichoderma koningiopsis* YIM PH30002 harbored in *Panax notoginseng*. Their structures were determined on the basis of spectral data interpretation. These compounds were evaluated for their antifungal activity, nitric oxide inhibition, and anticoagulant activity.

**Graphical Abstract:**

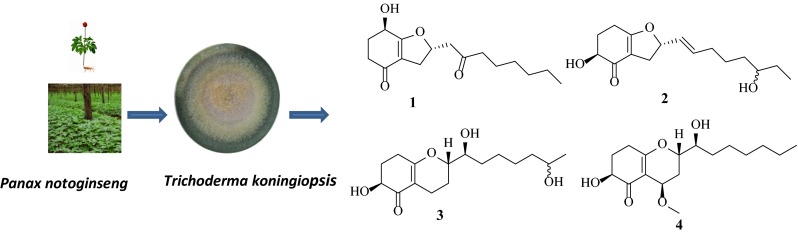

**Electronic supplementary material:**

The online version of this article (doi:10.1007/s13659-015-0085-z) contains supplementary material, which is available to authorized users.

## Introduction

**Fig. 1:**
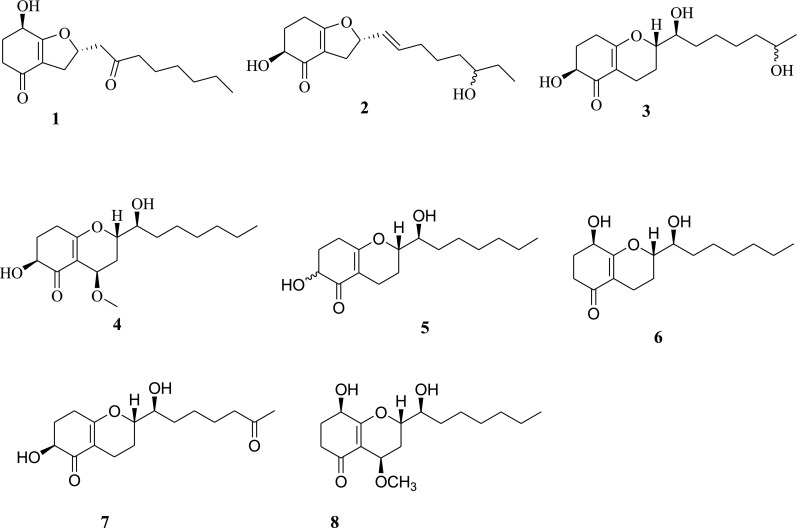
Chemical structures of compounds **1**–**8** from *T. koningiopsis* YIM PH30002

Endophytic microorganisms, residing in the living tissues of the host plant and forming complex relationships with the host, are to be found in every plant on earth. Species of *Trichoderma*, ubiquitous in the environment including plant and soil, produce a plethora of substances with potential use for modern agriculture, and industry [1, 2]. Some *Trichoderma* species can be used to enhancing plant growth and resistance to the biotic and abiotic stresses, such as antagonizing plant-pathogenic fungi, stimulating plant growth and defense responses, and inducing salt tolerance [3, 4]. At global level, *Trichoderma* species are widely used as a powerful biocontrol agent for plant diseases [5]. Chemical exploitation of *Trichoderma* spp. afforded a diverse of bioactive secondary metabolites [6–9]. Over the course of our ongoing search for new naturally occurring bioactive products from endophytic fungi associated with famous Chinese traditional medicine, *Panax notoginseng* [10], the chemical investigation of endophytic fungus *Trichoderma koningiopsis* YIM PH30002 was found with inhibiting the growth of some phytopathogenic fungi [11]. Four new metabolites named koninginins N-Q (**1**–**4**), together with four known koninginins B (**5**), E (**6**), J (**7**), and 7-*O*-methylkoninginin D (**8**) were isolated [9, 12–14] (Fig. 1). In the present paper, we report the isolation, structural elucidation and bioactivities of these fungal polyketides.

## Results and Discussion

**Fig. 2:**
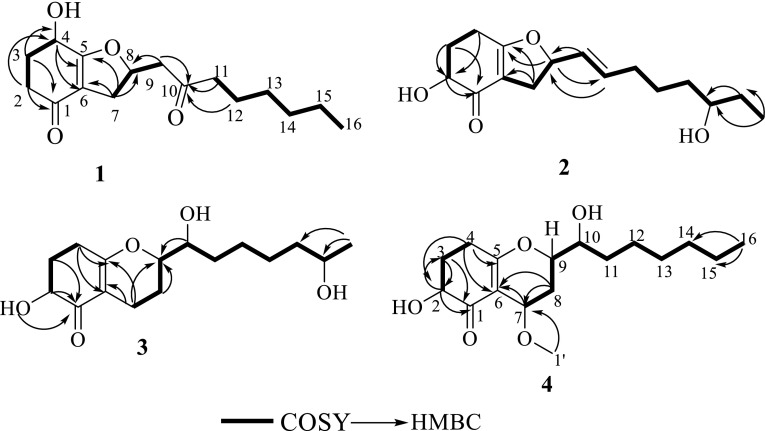
Key ^1^H- ^1^H COSY and HMBC correlations of compounds **1**–**4**

**Fig. 3:**
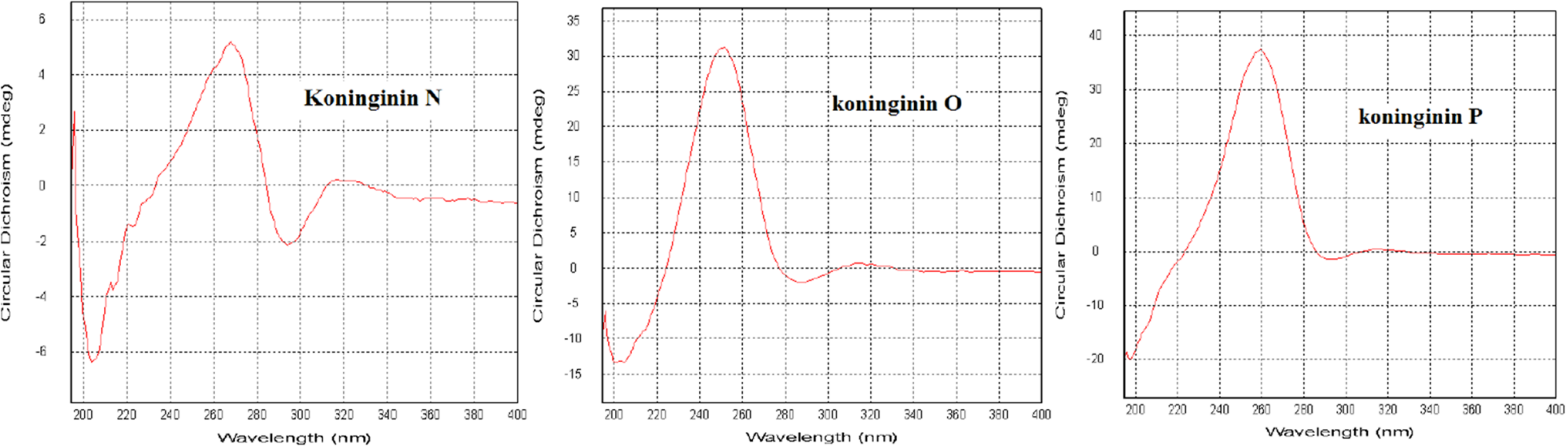
The CD spectra of compounds **1**–**3**

Compound **1** was obtained as oil. The molecular formula of **1** was determined to be C_16_H_24_O_4_ by analysis of its HRESI-MS spectrum and NMR data (Table 1). A detailed analysis of its NMR data revealed that its chemical structure was highly similar to trichodermaketone C [9]. The OH group connected to C-4 was determined by the correlations from H-4 to C-5, and C-6 in the HMBC spectrum and the correlation from H-2 to H-4 in the ^1^H-^1^H COSY spectrum. The connection of C-5 to C-8 by an oxygen atom was determined by the HMBC correlations from H-7 to C-5, 6 and the COSY correlation from H-7 to H-9. The carbonyl was placed at C-10 according to the correlations from H-9, 11, 12 to C-10 in the HMBC spectrum (Fig. 2). The relative stereochemistry at H_4_-H_8_ of compound **1** ([α] = −68.3) was determined to the same as trichodermaketone C by comparing the NMR data and optical rotation value of trichodermaketone C ([α] = −92.5) [9]. A negative Cotton effect at 292 nm (Δε −2.023) for the n → π* transition in the circular dichroism (CD) spectrum (Fig. 3) of **1** suggested that the absolute configuration of C-4 was 4R.

**Table 1:** ^1^H and ^13^C NMR Data of **1**–**4** in CDCl_3_ (*δ* in ppm, *J* in Hz)

Position	**1** ^a^		**2** ^b^		**3** ^b^		**4** ^a^	
	*δ*_H_	δ_c_	*δ*_H_	*δ*_c_	*δ*_H_	*δ*_c_	*δ*_H_	*δ*_c_
1		194.9		195.2		198.5		197.7
2	2.44, 2.33 (m)	35.0	4.10 (dd, 5.6)	71.9	4.07 (dd, 5.6)	71.4	4.15 (dd, 5.2)	71.0
3	2.31, 2.03 (m)	31.3	2.42, 1.89 (m)	30.7	2.36, 1.79 (m)	29.4	2.37, 1.78 (m)	29.2
4	4.57 (q, 6.4)	64.1	2.56 (m)	23.4	2.61, 2.48 (m)	27.6	2.64, 2.47 (m)	27.3
5		175.0		178.3		171.6		173.1
6		113.4		110.8		109.6		110.5
7	3.06, 2.44 (m)	32.5	3.09, 2.56 (m)	32.2	2.61, 2.09 (m)	18.1	4.39 (m)	65.4
8	5.28 (m)	82.2	5.28 (m)	87.9	1.95, 1.59 (m)	23.1	2.04, 1.63 (m)	28.1
9	2.97, 2.72 (m)	48.8	5.59 (m)	128.5	3.76 (m)	81.2	4.13 (m)	77.0
10		207.9	5.82 (m)	135.9	3.69 (m)	73.5	3.65 (m)	72.9
11	2.44 (m)	44.1	2.10 (m)	32.5	1.62 (m)	33.1	1.64 (m)	33.2
12	1.56 (m)	23.9	1.54 (m)	25.2	1.46 (m)	26.0	1.30 (m)	22.6
13	1.28 (m)	31.9	1.51 (m)	36.7	1.62 (m)	25.8	1.30 (m)	29.3
14	1.28 (m)	29.2	3.56 (m)	73.5	1.46 (m)	39.5	1.30 (m)	31.7
15	1.28 (m)	22.8	1.50 (m)	30.4	3.82 (m)	68.4	1.30 (m)	22.6
16	0.88 (t, 6.0)	14.4	0.94 (t, 7.6)	10.2	1.20 (d, 6.4)	24.0	0.90 (t, 6.5)	14.0
OCH_3_							3.38 (s)	56.9

^a^ ^1^H NMR was recorded at 400 MHz, and ^13^C NMR was recorded at 100 MHz

^b^ ^1^H NMR was recorded at 500 MHz, and ^13^C NMR was recorded at 125 MHz

The molecular formula of **2** was established as C_16_H_24_O_4_ by the HRESI-MS and NMR data. Compound **2** shared the same skeleton as compound **1** according to the NMR data (Table 1). The OH group connected to C-2 was confirmed by the HMBC correlation from H-2 to C-1 and the COSY correlations from H-2 to H-4. The connections of C-5 to C-8 by an oxygen atom and C_9_-C_10_ to C-8 were determined by the correlations from H-8 to C-5, H-7 to C-5 and C-6 in HMBC spectrum, and the correlations from H-7 to H-10 in ^1^H-^1^H COSY spectrum. Another OH group was assigned to C-14 by the HMBC correlations from H-16 to C-14, C-15 (Fig. 2). The presence of a *trans*-double bond was revealed by the corresponding carbon signals at *δ*_C_ 128.5 (C-9) and 135.9 (C-10). The absolute configurations of compound **2** in H-2 and H-8 were determined the same as these of trichoketide B by comparing the CD spectrum (Fig. 3), the NMR data and the optical rotation value of trichoketide B [15].

Compound **3** was isolated as pale-yellow oil. The molecular formula of **3** was determined to be C_16_H_26_O_5_ by its HRESI-MS and NMR data. After detailed analysis of the ^1^H and ^13^C NMR data (Table 1), the skeleton of compound **3** was elucidated to be the same as compound **7**. The OH group connected to C-2 was confirmed by the HMBC correlations from H-2 to C-1 and the COSY correlations from H-2 to H-4. The connection of C-5 to C-9 by the oxygen atom and OH connected to C-10 were determined by the COSY correlations from H-7 to H-10 and HMBC correlations from H-7 to C-5, C-6, C-9, and H-10 to C-9. The OH group connection to C-15 was confirmed by the HMBC correlation from H-16 to C-14, C-15 and COSY correlations from H-16 to H-15 (Fig. 2). In the ^1^H NMR spectrum, a coupling constant (*J* = 5.6 Hz) was observed for H-2, which required a *trans*-diequatorial relationship between the oxymethine proton (H-2) and H-3α. The absolute configurations of compound **3** in H-9, and H-10 were determined to be the same as that of koninginin B also isolated from one strain of *T*. *koningiopsis* [12] by the comparison of NMR spectra, CD spectrum (Fig. 3), and their biogenesis.

The molecular formula of **4** was elucidated as C_17_H_28_O_5_ by analysis of its HRESI-MS spectrum and NMR data (Table 1). A detailed analysis of its NMR data revealed a highly similar structure to koninginin F [12]. The full assignments of its ^1^H and ^13^C NMR (Table 1) were obtained by 2D NMR (Fig. 2). Two oxygen atoms were assigned to C-7 and C-9, respectively, according to HMBC correlations from H-7 to C-6, and the COSY correlations from H-7 to H-9. The OH group at C-2 was determined by the correlation between H-2 and C-1 in the HMBC spectrum, and another OH group was placed at C-10 on the basis of the cross peak of H-9 to H-10 in the ^1^H-^1^H COSY spectrum. The methoxy group was elucidated by the correlation from OCH_3_ to C-7 in the HMBC spectrum. In the ^1^H NMR, a coupling constant (*J* = 5.2 Hz) was observed for H-2, which required a *trans*-diequatorial relationship between the oxymethine proton (H-2) and H-3α. The stereochemistry of C-7, C-9, and C-10 in compound **4** was the same as that of koninginin F also isolated from one strain of *T*. *koningiopsis* by the NMR comparison, CD spectrum (Fig. 3), and their biogenesis [12].

The absolute configurations of **2**–**4** were assigned as 2S, similar to the known compounds, on the bases of CD spectral analyses and their biosynthetic pathway (Fig. 3) [8, 15].

The antifungal activities of compounds **1–8** were challenged with the phytopathogenic fungi, *Fusarium oxysporum, F. solani*, *F. flocciferum*, *Plectosphaerella cucumerina*, *Alternaria panax* which are causes of root rot diseases of *P*. *notoginseng* [16]. Koninginin O (**2**) and koninginin Q (**5**) showed the weak activity against *F. oxysporum*, *P. cucumerina*, with an MIC of 128 µg/mL. 7-*O*-methylkoninginin D (**8**) showed weak activity against *P. cucumerina* with an MIC of 128 µg/mL. Other compounds present no antifungal activity as their MICs > 128 µg/mL. The positive control compound, nystatin showed antifungal activity with MICs at 32 μg/mL. Compound **1** showed low active in the NO inhibitory tests with IC_50_ > 25 μM (MG 132 at 0.18 μM), and compound **5** showed no obvious anticoagulant activity with APTT at 39.8 ± 0.42 s (LMWH at 135.8 ± 0.85 s).

## Experimental Section

### General

Optical rotations were measured on a Perkin-Elmer 341 automatic polarimeter (Perkin-Elmer). UV spectra were recorded in MeOH (1 mg/50 mL) on a UV 210A spectrophotometer (Shimadzu). NMR spectra were recorded on a Bruker DRX-500 and DRX-400 spectrometer (Bruker). The chemical shifts (*δ*) are reported in ppm using tetramethylsilane as an internal standard and the coupling constants (*J*) are given in Hertz (Hz). HRESI-MS spectra were recorded on Agilent G3250AA (Agilent) and AutoSpec Premier P776 spectrometers (Waters). Column chromatography (CC) was performed on self-packed open columns with silica gel from Qingdao Haiyang Chemical Co., Ltd (QHCC). Thin layer chromatography (TLC) analyses were conducted on glass sheets of silica gel GF_254_ from QHCC and detected under a UV lamp at 254 or 365 nm and visualized by spraying 8 % phosphomolybdic acid-EtOH solution (w/v) or 5 % vanillin-H_2_SO_4_ (w/v) followed by heating, or visualized with iodine (I_2_). Semi-preparative high performance liquid chromatography (HPLC) (Agilent 1200 Series) was performed on an YMC C_18_ column (250 mm × 10 mm, 5 μm, YMC Karasuma-Gojo Bldg.). Fractions from all columns were collected by an auto-collecting apparatus and were combined according to TLC analyses. All other solvents and reagents were commercially purchased from Beijing Greenherbs Science and Technology Development Co., Ltd. and distilled prior to use.

### Fungi Strain and Materials

The endophytic fungus *T*. *koningiopsis* YIM PH30002 was isolated from a 2-year-old healthy *P. notoginseng* plant collected from Wenshan, Yunnan Province, China, in March 2012. Potato Dextrose Agar (PDA) medium (infusion of 200 g fresh potato, dextrose 15 g, and 1 L distilled water, agar 15.0 g, pH 7.0) was used for isolation of the fungus and maintenance of the culture for identification purposes. The stock culture of *T*. *koningiopsis* YIM PH30002 was grown on the PDA slant at 4 °C. Microbial identification was performed with rDNA-ITS molecular-phylogenetic analysis and morphology characteristics in different growth stages. The BLAST sequenced data have been deposited at GenBank (Accession No. KM190127). A voucher specimen (No. YIM PH30002) was preserved at the Yunnan Institute of Microbiology, Kunming, China.

### Fermentation of *T. koningiopsis* YIM PH30002

*Trichoderma koningiopsis* YIM PH30002 was maintained on the fresh PDA medium. Small agar plugs (approximately 5 × 5 mm) with the fungus were cultured in 0.5-L Erlenmeyer flasks containing 100-mL potato dextrose broth (PDB, potato infusion of 200 g fresh potato, dextrose 15 g, distilled water 1.0 L, pH 7.0) at 130 rpm and 28 °C for 3 days. Large-scale culture was performed by transferring a-10 mL of seed culture into an 1-L Erlenmeyer flask containing 250 mL of PDB and incubated at 130 rpm and 28 °C for 10 days.

### Extraction and Isolation

The production culture (50 L) was centrifuged to separate mycelia from the supernatant. The supernatant was exhaustively extracted with EtOAc, yielding 30.6 g of extract. The mycelia were extracted three times with a mixture of 80 % acetone in H_2_O with ultrasonic processing (53 kHz, 180 W) for 15 min each time. Acetone was removed under vacuum, and the resulting aqueous layer (800 mL) was extracted three times with an equal volume of EtOAc to yield mycelial extract (13 g). The extracts of the fermentation broth and the mycelia were combined after TLC and HPLC analysis. The organic extract was repartitioned between petroleum ether and 10 % aqueous MeOH to remove lipids and pigments. The aqueous MeOH extract was dried under vacuum to yield extract (18.6 g). This extract was fractionated by column chromatography on silica gel (7 cm × 30 cm, 200–300 mesh, 0.6 kg, flow rate at 30 mL/min) eluting with a stepwise gradient using 2 L of CHCl_3_/MeOH at ratios of 1:0, 100:1, 50:1, 20:1, 10:1, 5:1, and 0:1 *v/v*, and a total of seven fractions (Fr. 1–Fr. 7) were collected. Fraction Fr. 1 (5 g) was eluted with a Sephadex LH-20 column (6 cm × 70 cm, methanol, 0.4 L each, flow rate at 7 mL/min) to give four sub-fractions (Fr. 1.1–Fr. 1.4). Compounds **1** (7.2 mg, t_R_ = 14.4 min) and **6** (2.6 mg, t_R_ = 23.0 min) were purified by semipreparative HPLC with gradient eluted with MeOH-H_2_O (3.0 mL/min, 50–80 %, 40 min) from sub-Fr. 1.1. Compound **8** (8.7 mg, t_R_ = 16.2 min) was purified by semipreparative HPLC with gradient eluted with MeOH-H_2_O (3.0 mL/min, 50–68 %, 40 min) from sub-Fr. 1.2. Fr.2 (8 g) was fractioned by column chromatography on MCI gel gradient eluted with MeOH-H_2_O (20–100 %) to give nine subfractions (Fr. 2.1–Fr. 2.9). Compounds **2** (2.2 mg, t_R_ = 17.8 min) and **5** (2.6 mg, t_R_ = 20.5 min) were purified by semipreparative HPLC with gradient eluted with MeOH-H_2_O (3.0 mL/min, 30–75 %, 45 min) from sub-Fr. 2.1. Compound **4** (2.4 mg, t_R_ = 18.4 min) was purified by semipreparative HPLC with gradient eluted with MeOH-H_2_O (3.0 mL/min, 35–65 %, 45 min) from sub-Fr. 2.2. The sub-fraction Fr. 2.4 (0.6 g) was eluted upon Sephadex LH-20 (methanol, 0.3 L each at a flow rate 4 mL/min) and further purified by semipreparative HPLC with gradient eluted with MeOH-H_2_O (3.0 mL/min, 35–50 %, 45 min) to afford compounds **3** (3.3 mg, t_R_ = 18.6 min) and **7** (3.2 mg, t_R_ = 22.6 min).

#### Koninginin N (**1**)

Pale-yellow oil; $[\alpha ]_{{\text{D}}}^{{20}}$ −68.3 (*c* = 0.2, MeOH); UV (MeOH) λ_max_ (log *ε*) 250 nm (4.12); ^1^H and ^13^C NMR data, see Table 1; HRESI-MS *m/z*: 303.1579 [M + Na]^+^ (calcd for C_16_H_24_O_4_Na 303.1572).

#### Koninginin O (**2**)

Pale-yellow oil; $[\alpha ]_{{\text{D}}}^{{20}}$ 1.6 (*c* = 0.1, MeOH); UV (MeOH) λ_max_ (log *ε*) 253 nm (3.91); ^1^H and ^13^C NMR data, see Table 1; HRESI-MS *m/z*: 303.1569 [M + Na]^+^ (calcd for C_16_H_24_O_4_Na 303.1572).

#### Koninginin P (**3**)

Pale-yellow oil; $[\alpha ]_{{\text{D}}}^{{20}}$ −3.8 (*c* = 0.1, MeOH); UV (MeOH) λ_max_ (log *ε*) 256 nm (4.05); ^1^H and ^13^C NMR data, see Table 1; HRESI-MS *m/z*: 321.1675 [M + Na]^+^ (calcd for C_16_H_26_O_5_Na 321.1678).

#### Koninginin Q (**4**)

Pale-yellow oil; $[\alpha ]_{{\text{D}}}^{{20}}$ 36.1 (*c* = 0.2, MeOH); UV (MeOH) λ_max_ (log *ε*) 256 nm (3.87); ^1^H and ^13^C NMR data, see Table 1; HRESI-MS *m/z*: 335.1838 [M + Na]^+^ (calcd for C_17_H_28_O_5_Na 335.1834).

### Antimicrobial Activity, NO Inhibitory Activity, and Anticoagulant Activity Assays

The antimicrobial activities of compounds **1**–**8** were evaluated with the methods described in NCCLS standard M27-A2 and M7-A7 [17, 18]. Nystatin (Taicheng Pharmaceutical Co., Ltd., purity >99 %) were employed as positive controls in the antifungal assays, respectively. All experiments were treated in triplicate. The microbial growth was observed with a CX21BIM-set5 microscope (Olympus Corp). MIC was determined as the lowest concentration that produced complete growth inhibition of the tested microorganism.

The NO inhibitory activity of **1** was determined using the Griess reagent assay for NO production [19]. Briefly, the murine macrophage cell line was used as detection model. The supernatants were used to measure the NO production with an MTT [3-(4,5-dimethylthiazol-2-yl)-2,5-diphenyltetrazoliumbromide] assay for cell viability. MG132 (proteasome inhibitor) was used as the positive control.

The in vitro anticoagulant activity of compound **5** was investigated by the method of activated partial thromboplastin time (APTT) [20]. Low molecular weight heparin (LMWH) was used as the positive control.

## Electronic supplementary material

Supplementary material 1 (DOCX 6641 kb)
