# Supplementary material for: Koninginins N-Q, Polyketides from the Endophytic Fungus Trichoderma koningiopsis Harbored in Panax notoginseng
Source: Nat Prod Bioprospect. 2016 Jan 11;6(1):49–55. doi: 10.1007/s13659-015-0085-z (PMC4749524; doi:10.1007/s13659-015-0085-z)
Supplement: Supplementary file 1 — Supplementary material 1 (DOCX 6641 kb) [file 13659_2015_85_MOESM1_ESM.docx]

Supporting Information

**Koninginins N-Q, Polyketides from the Endophytic Fungus *Trichoderma koningiopsis* Harbored in *Panax notoginseng***

Kai Liu^a^, Yabin Yang^b^, Jin-Lian Chen^c^, Cui-Ping Miao^a^, Qiang Wang^a^, Hao Zhou^b^, You-Wei Chen^a^, Yi-Qing Li^a^, Zhong-Tao Ding^b^, Li-Xing Zhao^a^

^a^ Yunnan Institute of Microbiology, School of Life Science, Yunnan University, Kunming 650091, China

^b^Key Laboratory of Medicinal chemistry for Natural Resource, Ministry of Education, School of Chemical Science and Technology, Yunnan University, Kunming 650091, P.R. China

^c^School of Energy and Environment Science, Yunnan Normal University, Kunming 650092, P. R. China

Correspondence

Dr. Li-Xing Zhao, Yunnan Institute of Microbiology, Yunnan University, Kunming 650091, People’s Republic of China. E-mail: zlx70@163.com. Phone: 86-871-65033539

Prof. Dr. Zhongtao Ding, School of Chemical Science and Technology, Yunnan University, Kunming 650091, People’s Republic of China. E-mail: ztding@ynu.edu.cn Phone: 86-871-65033910

Figure S1. ^1^H NMR Spectrum of koninginin N (1) in CDCl_3_……………………….3

Figure S2. ^13^C NMR Spectrum of koninginin N (1) in CDCl_3_....................................3

Figure S3. ^1^H-^1^H COSY Spectrum of koninginin N(1) in CDCl_3……………………………._.4

Figure S4. HSQC Spectrum of koninginin N (1) in CDCl_3_………………………….4

Figure S5. HMBC Spectrum of koninginin N (1) in CDCl_3_…………………………..5

Figure S6. ROESY Spectrum of Koningnin N (1) in CDCl_3_………………………...5

Figure S7. HRESIMS Spectrum of koninginin N (1)………………………………. .6

Figure S8. ^1^H NMR Spectrum of koninginin O (2) in CDCl_3_………………………..6

Figure S9. ^13^C NMR Spectrum of koninginin O (2) in CDCl_3_……………………….7

Figure S10. ^1^H-^1^H COSY Spectrum of koninginin O(2) in CDCl_3_………………… 7

Figure S11. HSQC Spectrum of koninginin O (2) in CDCl_3_…………………… …8

Figure S12. HMBC Spectrum of koninginin O (2) in CDCl_3_,………………………. .8

Figure S13. ROESY Spectrum of Koningnin O (2) in CDCl_3_…………………… …9

Figure S14. HRESIMS Spectrum of koninginin O (2)……………………………… 9

Figure S15. ^1^H NMR Spectrum of koninginin P (3) in CDCl_3_………………………10

Figure S16. ^13^C NMR Spectrum of koninginin P (3) in CDCl_3_……………………. .10

Figure S17. ^1^H-^1^H COSY Spectrum of koninginin P(3) in CDCl_3_…………………. 11

Figure S18. HSQC Spectrum of koninginin P (3) in CDCl_3_…………………….. 11

Figure S19. HMBC Spectrum of koninginin P (3) in CDCl_3_………………………12

Figure S20. ROESY Spectrum of Koningnin P (3) in CDCl_3_…………………… …12

Figure S21. HRESIMS Spectrum of koninginin P (3).................................................13

Figure S22. ^1^H NMR Spectrum of koninginin Q (4) in CDCl_3_……………………..13

Figure S23. ^13^C NMR Spectrum of koninginin Q (4) in CDCl_3_……………….……14

Figure S24. ^1^H-^1^H COSY Spectrum of koninginin Q (4) in CDCl_3_…………………14

Figure S25. HSQC Spectrum of koninginin Q (4) in CDCl_3_……………………… .15

Figure S26. HMBC Spectrum of koninginin Q (4) in CDCl_3_………………………..15

Figure S27. ROESY Spectrum of Koningnin Q (4) in CDCl_3_……………………... 16

Figure S28. HRESIMS Spectrum of koninginin Q (4)……………………………. .16


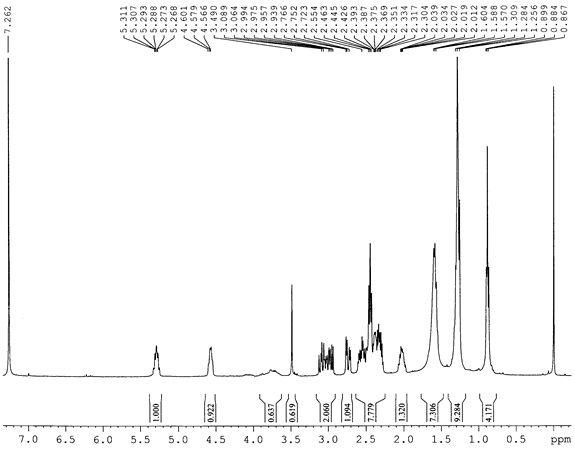


Figure S1. ^1^H NMR Spectrum of koninginin N (1) in CDCl_3_


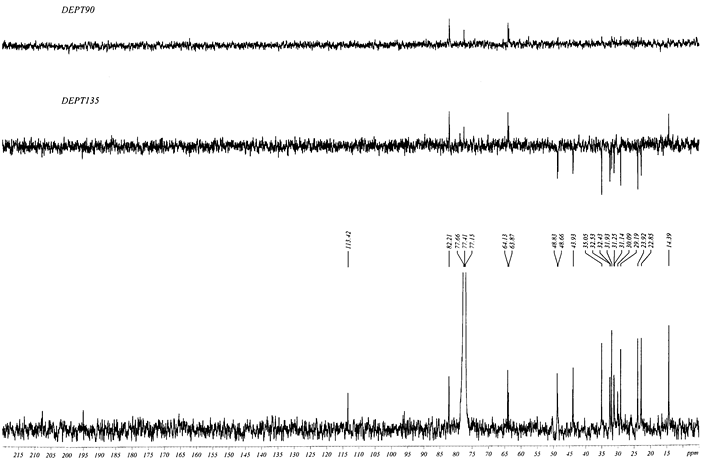


Figure S2. ^13^C NMR Spectrum of koninginin N (1) in CDCl_3_


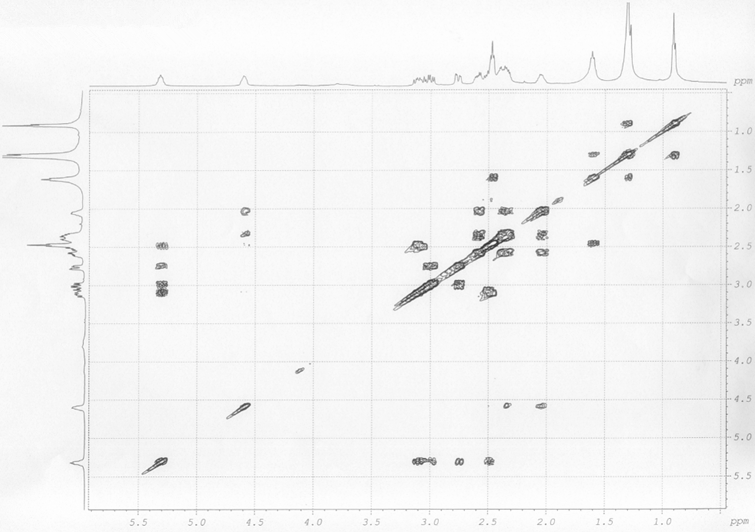


Figure S3. ^1^H-^1^H COSY Spectrum of koninginin N(1) in CDCl_3_


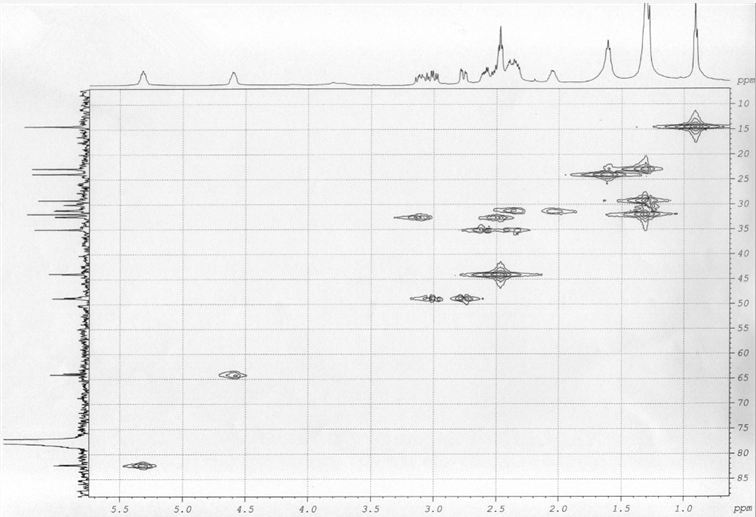


Figure S4. HSQC Spectrum of koningininN (1) in CDCl_3_


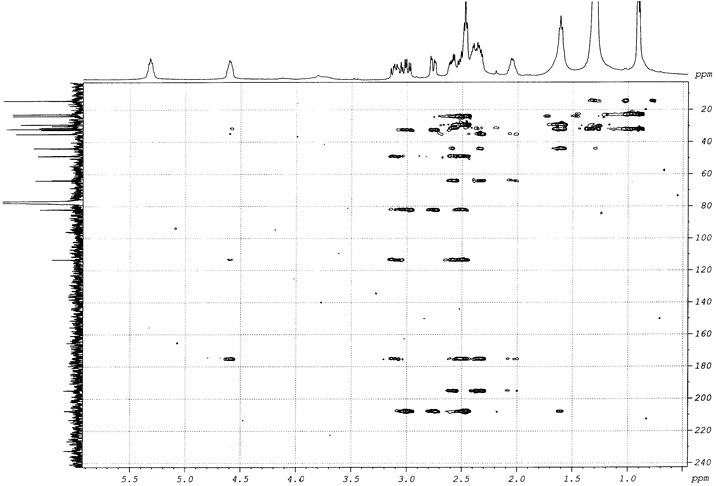


Figure S5. HMBC Spectrum of koninginin N (1) in CDCl_3_


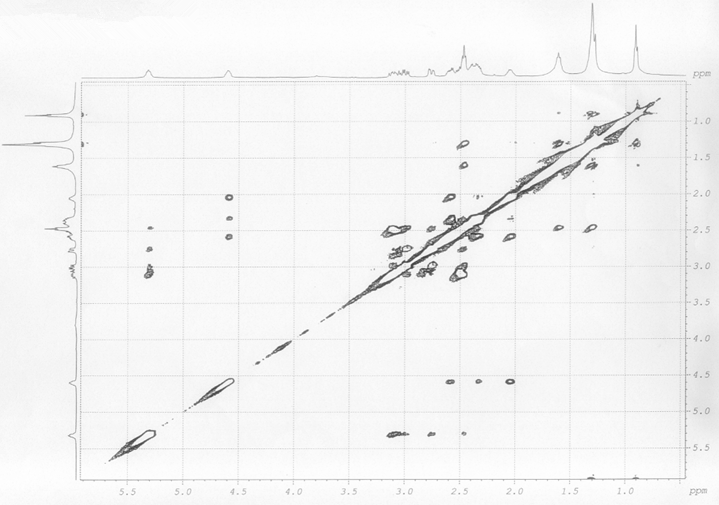


Figure S6. ROESY Spectrum of Koningnin N (1) in CDCl_3_


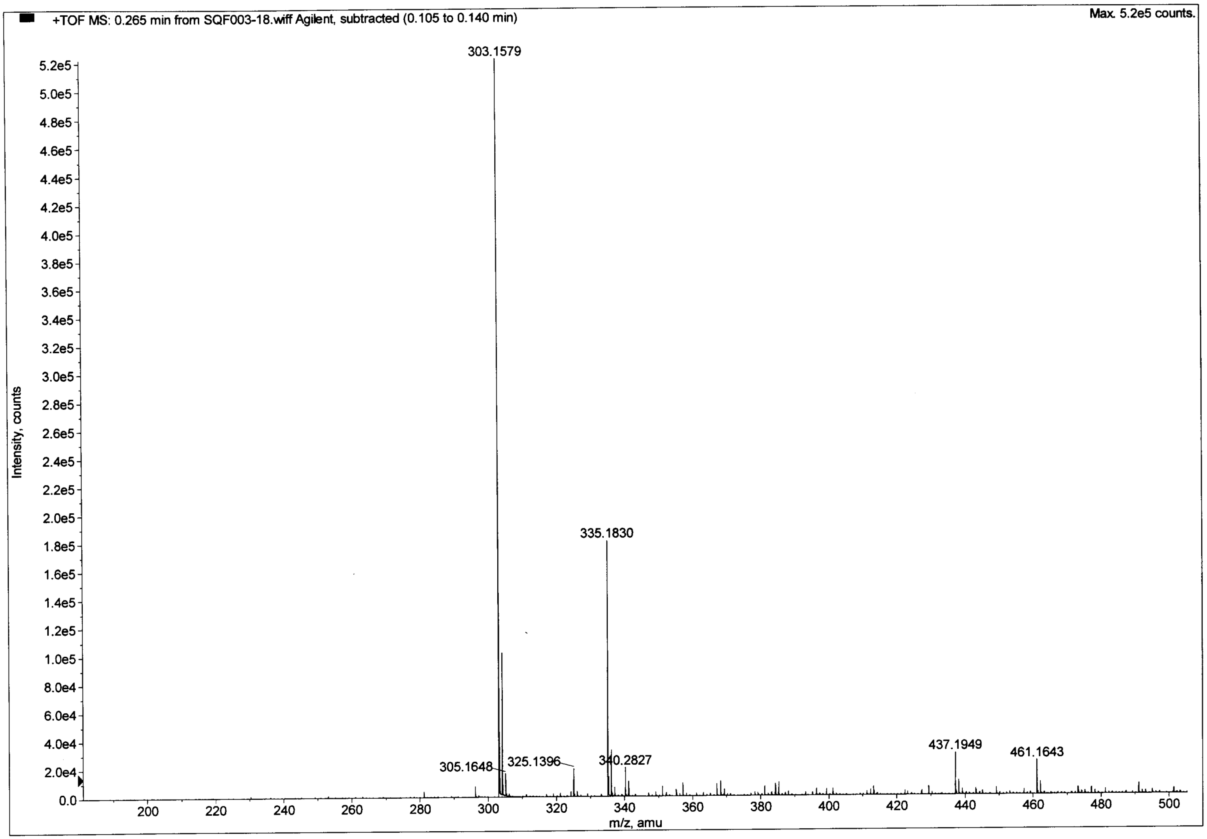


Figure S7. HRESIMS Spectrum of koninginin N (**1**)


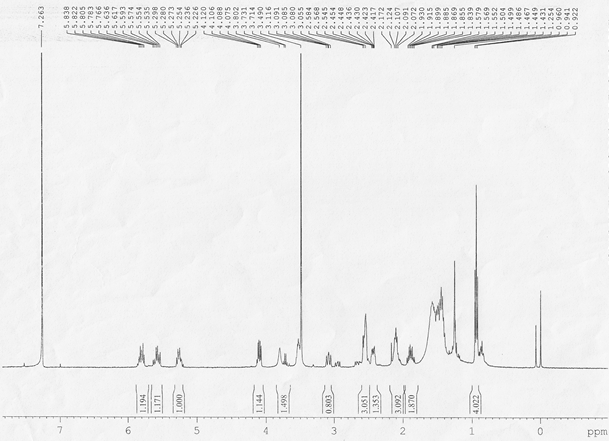


Figure S8. ^1^H NMR Spectrum of koninginin O (2) in CDCl_3_


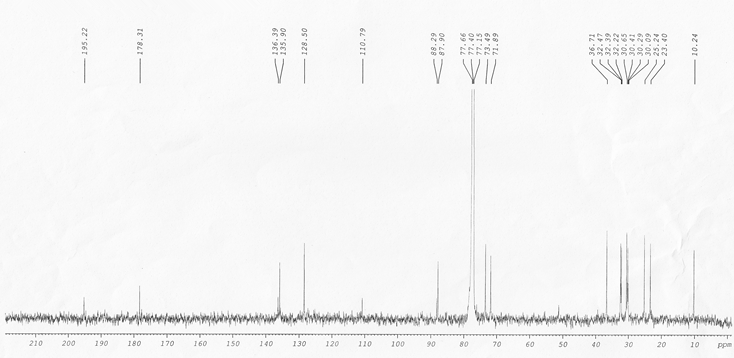


Figure S9. ^13^C NMR Spectrum of koninginin O (2) in CDCl_3_


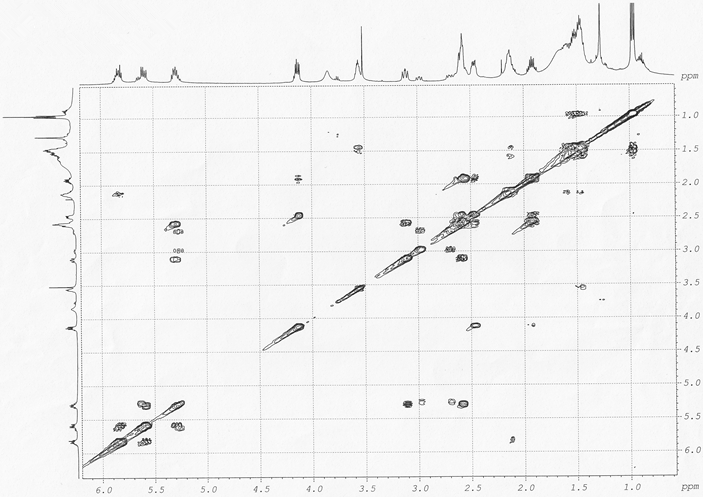


Figure S10. ^1^H-^1^H COSY Spectrum of koninginin O(2) in CDCl_3_


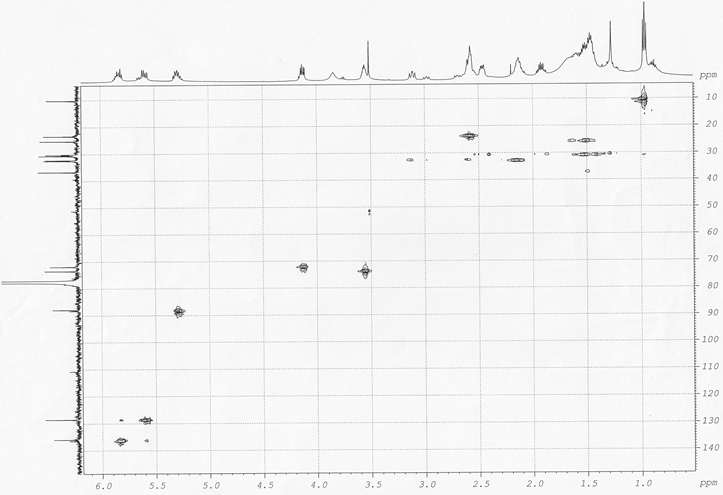


Figure S11. HSQC Spectrum of koninginin O (2) in CDCl_3_


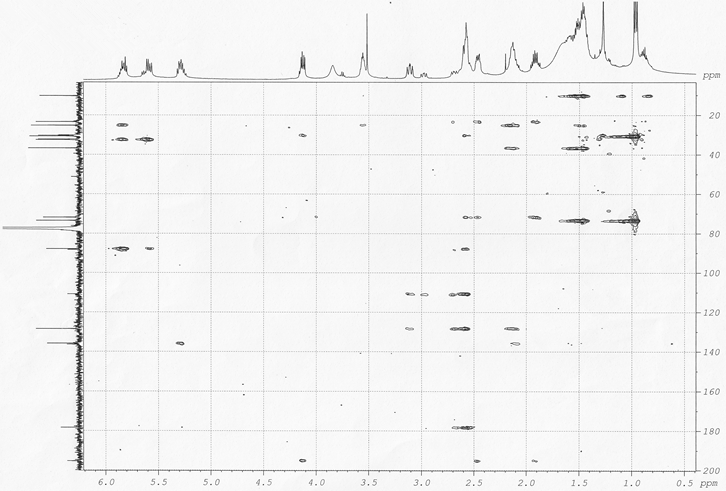


Figure S12. HMBC Spectrum of koninginin O (2) in CDCl_3_


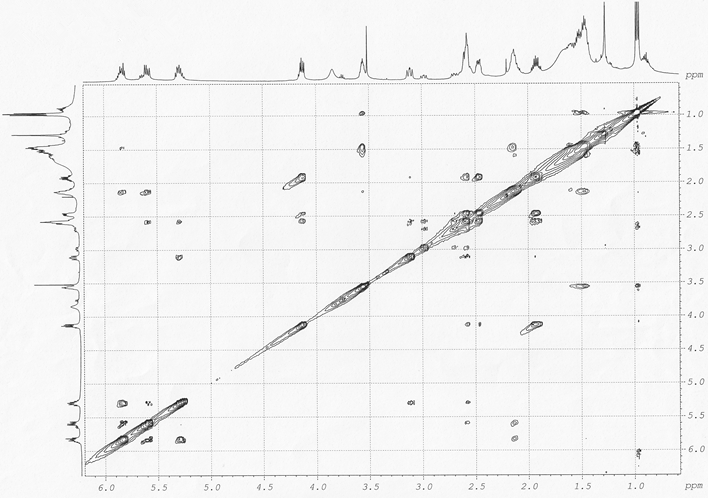


Figure S13. ROESY Spectrum of Koningnin O (2) in CDCl_3_


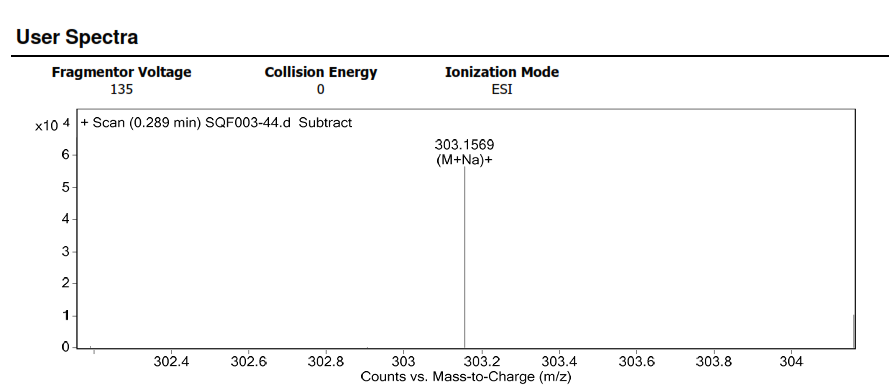


Figure S14. HRESIMS Spectrum of koninginin O (**2**)


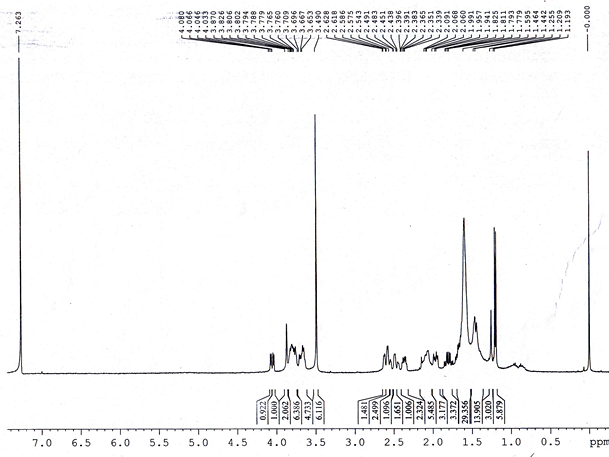


Figure S15. ^1^H NMR Spectrum of koninginin P (3) in CDCl_3_


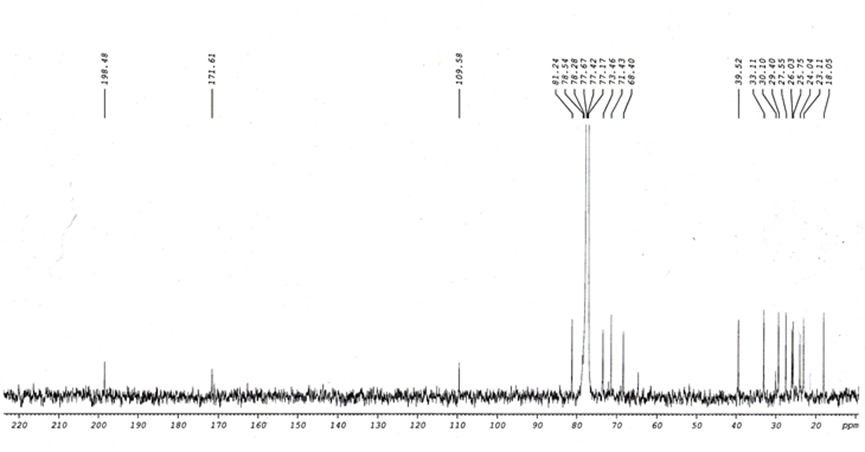


Figure S16. ^13^C NMR Spectrum of koninginin P (3) in CDCl_3_


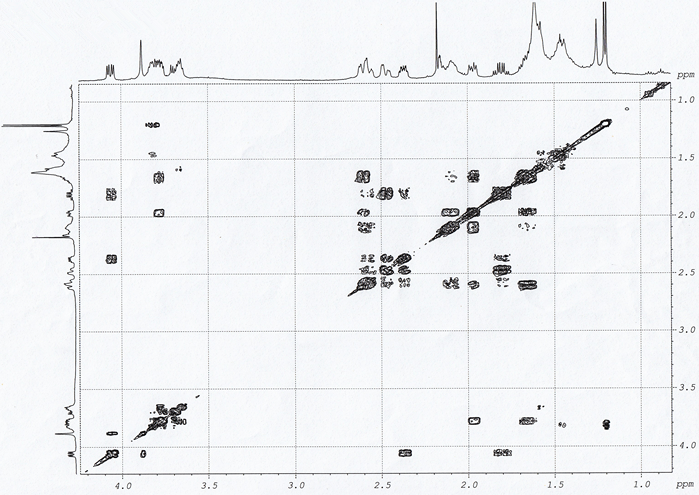


Figure S17. ^1^H-^1^H COSY Spectrum of koninginin P(3) in CDCl_3_


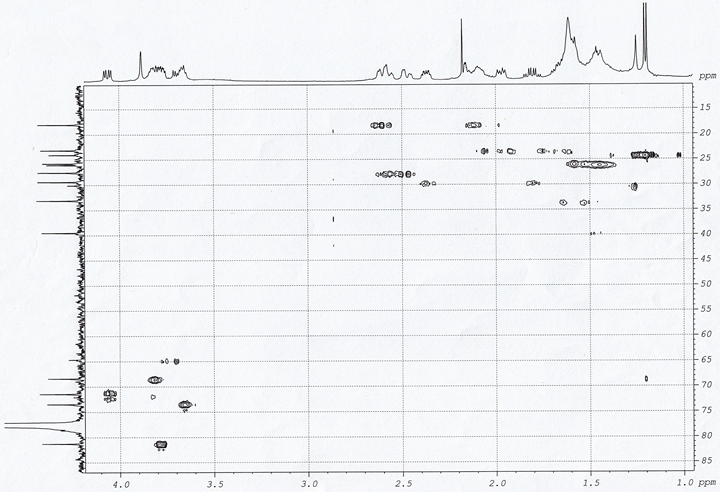


Figure S18. HSQC Spectrum of koninginin P (3) in CDCl_3_


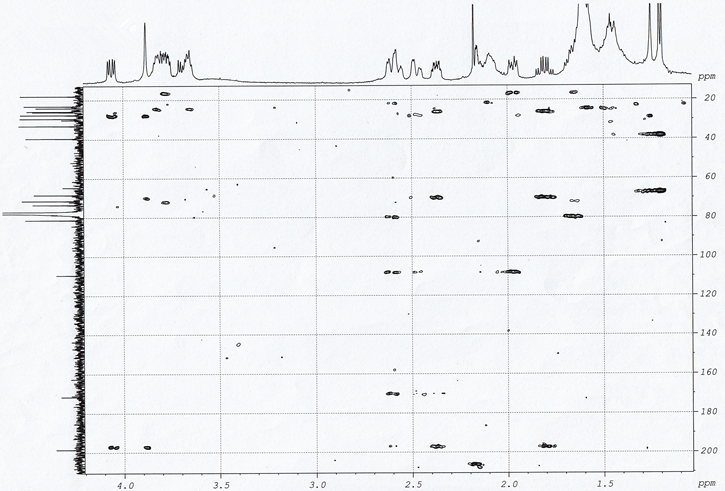


Figure S19. HMBC Spctrum of koninginin P (3) in CDCl_3_


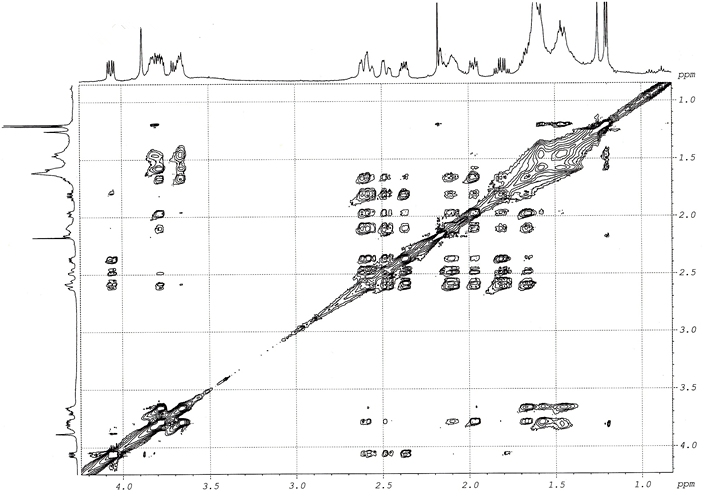


Figure S20. ROESY Spectrum of Koningnin P (3) in CDCl_3_


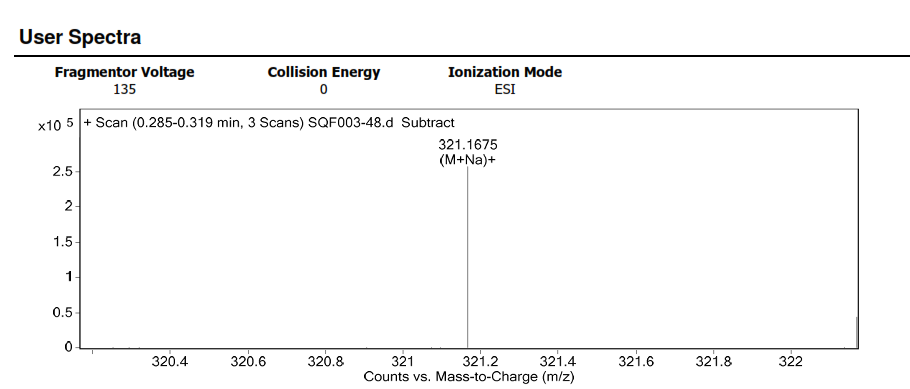


Figure S21. HRESIMS Spectrum of koninginin P (**3**)


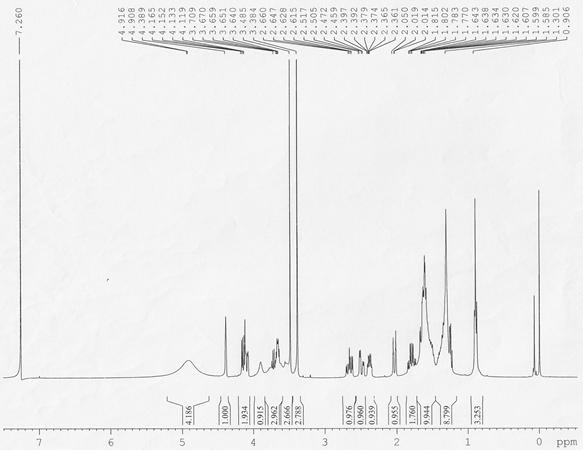


Figure S22. ^1^H NMR Spectrum of koninginin Q (**4**) in CDCl_3_


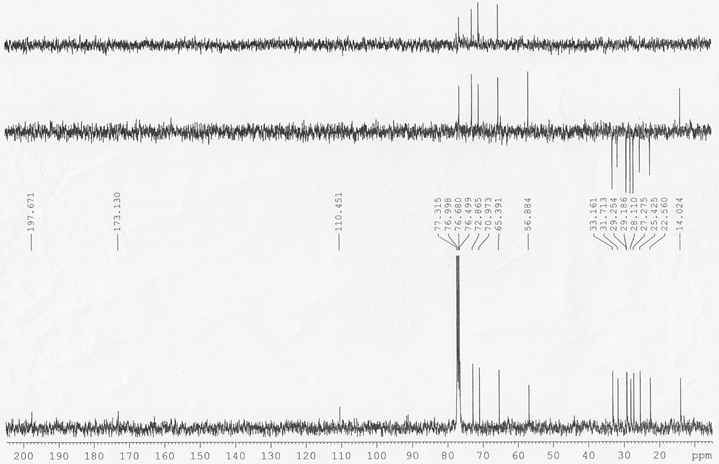


Figure S23. ^13^C NMR Spectrum of koninginin Q (4) in CDCl_3_


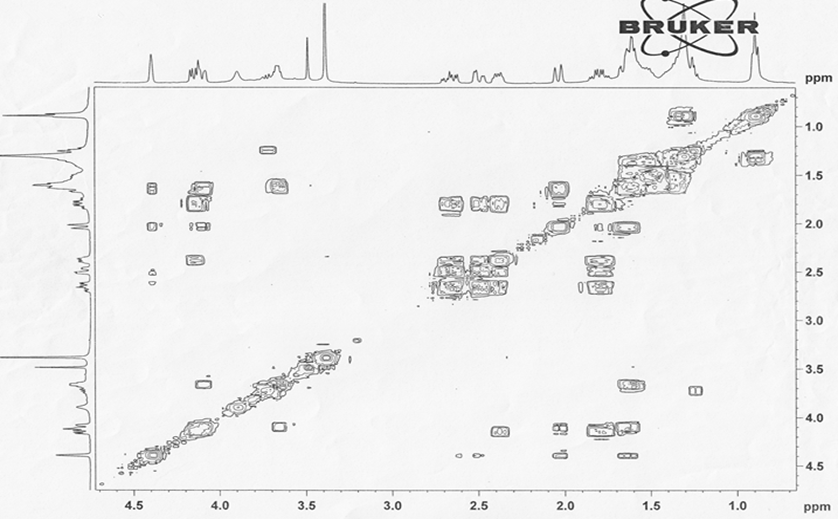


Figure S24. ^1^H-^1^H COSY Spectrum of koninginin Q (4) in CDCl_3_


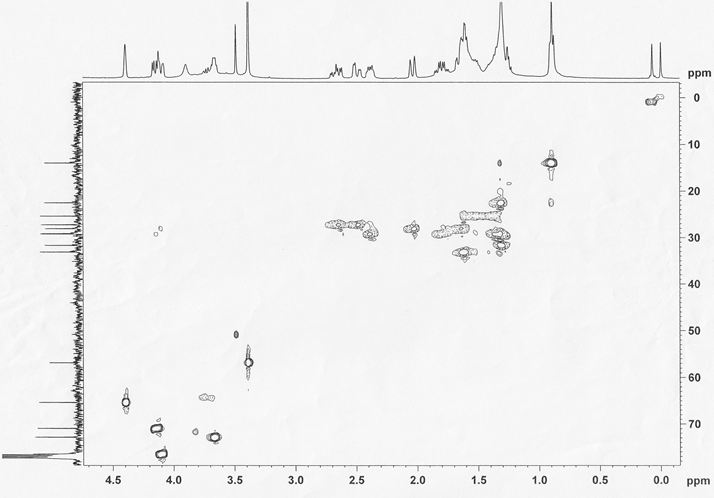


Figure S25. HSQC Spectrum of koninginin Q (4) in CDCl_3_

Figure S26. HMBC Spectrum of koninginin Q (4) in CDCl_3_


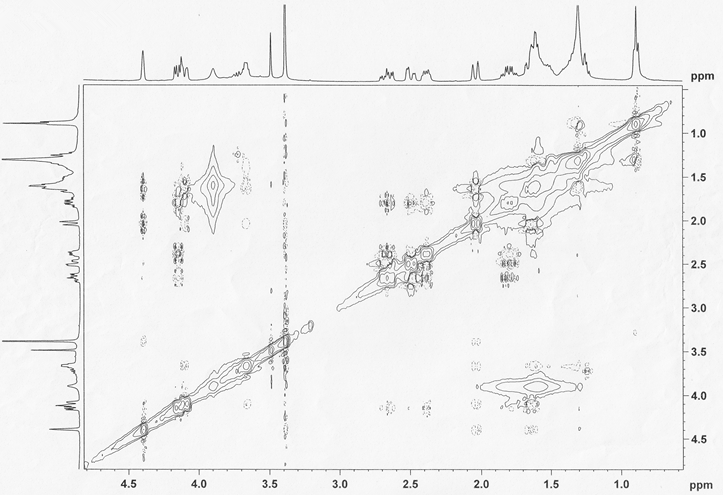


Figure S27. ROESY Spectrum of Koningnin Q (4) in CDCl_3_


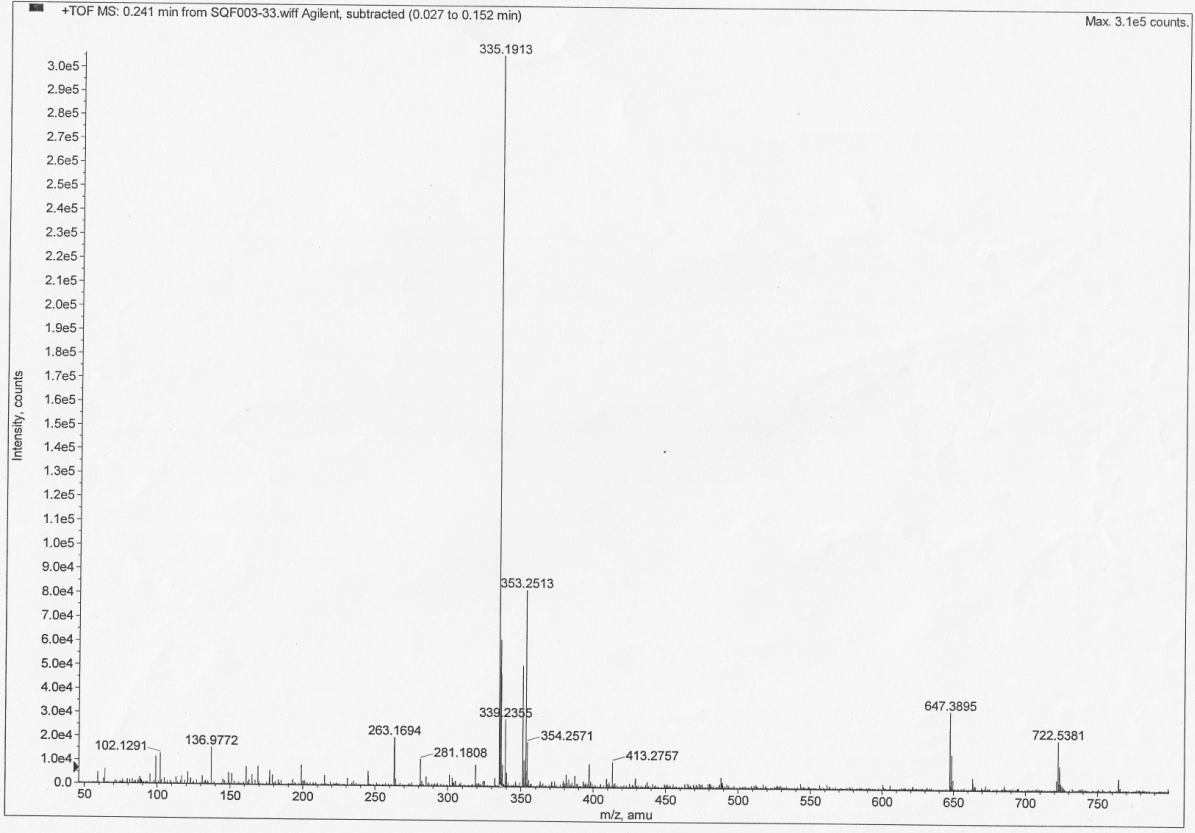


Figure S28. HRESIMS Spectrum of koninginin Q (**4**)
